# Consistency-Driven Dual-Teacher Framework for Semi-Supervised Zooplankton Microscopic Image Segmentation

**DOI:** 10.3390/jimaging12030125

**Published:** 2026-03-12

**Authors:** Zhongwei Li, Yinglin Wang, Dekun Yuan, Yanping Qi, Xiaoli Song

**Affiliations:** 1 College of Oceanography and Space Informatics, China University of Petroleum (East China), Qingdao 266580, China; 2 North China Sea Environmental Monitoring Center, State Oceanic Administration, Qingdao 266033, China; qiyanping@ncs.mnr.gov.cn (Y.Q.);

**Keywords:** semantic segmentation, driven-consistency learning, pseudo-label supervision, multi-teacher collaboration, marine plankton imaging

## Abstract

In-depth research on marine biodiversity is essential for understanding and protecting marine ecosystems, where semantic segmentation of marine species plays a crucial role. However, segmenting microscopic zooplankton images remains challenging due to highly variable morphologies, complex boundaries, and the scarcity of high-quality pixel-level annotations that require expert knowledge. Existing semi-supervised methods often rely on single-model perspectives, producing unreliable pseudo-labels and limiting performance in such complex scenarios. To address these challenges, this paper proposes a consistency-driven dual-teacher framework tailored for zooplankton segmentation. Two heterogeneous teacher networks are employed: one captures global morphological features, while the other focuses on local fine-grained details, providing complementary and diverse supervision and alleviating overfitting under limited annotations. In addition, a dynamic fusion-based pseudo-label filtering strategy is introduced to adaptively integrate hard and soft labels by jointly considering prediction consistency and confidence scores, thereby enhancing supervision flexibility. Extensive experiments on the Zooplankton-21 Microscopic Segmentation Dataset (ZMS-21), a self-constructed microscopic zooplankton dataset demonstrate that the proposed method consistently outperforms existing semi-supervised segmentation approaches under various annotation ratios, achieving mIoU scores of 64.80%, 69.58%, 70.32%, and 73.92% with 1/16, 1/8, 1/4, and 1/2 labeled data, respectively.

## 1. Introduction

The ocean is a major reservoir of biodiversity. Due to their high sensitivity to environmental changes in terms of species composition, spatial distribution, and population abundance, marine zooplankton serve as key indicator organisms for assessing the health of marine ecosystems. Accurate identification of different zooplankton species is therefore a fundamental task in marine biodiversity research. Traditionally, zooplankton identification relies on manual analysis of microscopic images and expert-driven annotation and classification, which are labor-intensive, time-consuming, and difficult to scale. With the increasing demand for large-scale and long-term biodiversity monitoring, the development of automated zooplankton identification methods has become increasingly urgent.

In recent years, advances in microscopy and artificial intelligence have enabled new possibilities for automated zooplankton recognition. Nevertheless, intelligent recognition techniques for zooplankton diversity remain at an early stage of development. Among them, semantic segmentation plays a crucial role as a prerequisite for fine-grained recognition, as it assigns a semantic label to each pixel and enables precise delineation of zooplankton morphologies while effectively separating adjacent or overlapping individuals. Although semantic segmentation has been widely and successfully applied in medical imaging and autonomous driving, its application to zooplankton microscopy images faces several unique challenges. Zooplankton exhibit highly diverse morphologies, complex and irregular boundaries, and intricate fine structures such as antennae and appendages, which significantly increase segmentation difficulty. Moreover, pixel-level annotations typically require extensive expertise from marine biologists and meticulous manual effort, making large-scale annotation costly and time-consuming. These factors severely limit the scalability of fully supervised segmentation methods for zooplankton studies.

To address the performance degradation caused by limited annotations, semi-supervised semantic segmentation has emerged as a more practical alternative by jointly leveraging a small set of labeled samples and a large amount of unlabeled data. Although fully unsupervised segmentation methods eliminate the need for manual annotations, they often struggle to establish reliable semantic correspondences in complex biological imaging scenarios. In zooplankton microscopic images, different species may share highly similar local structures while exhibiting subtle global morphological differences. Many unsupervised approaches construct supervisory signals by relying on clustering-based objectives [1,2], self-supervised feature learning followed by dense grouping or representation refinement [3,4,5], or distribution alignment mechanisms that match feature or output distributions without explicit target labels [6,7]. Since these paradigms do not incorporate human-provided semantic anchors for predefined biological categories, the resulting clusters or aligned representations may not consistently correspond to species-level semantics, particularly under conditions of high inter-class similarity and fine-grained boundary requirements. In contrast, semi-supervised learning leverages a small set of expert-annotated pixel-level labels to provide reliable semantic guidance, while still exploiting abundant unlabeled data to enhance representation learning, making it more suitable for zooplankton segmentation tasks characterized by complex morphologies and high annotation costs.

Existing semi-supervised segmentation approaches mainly include consistency regularization based on spatial perturbations [8,9], self-training methods based on pseudo-label generation [10], and dual-learning strategies that integrate multi-scale features [11]. While these methods have achieved promising results on public benchmarks, they still exhibit notable limitations when applied to zooplankton microscopy images. Specifically, (1) most existing approaches rely on a single-teacher model, which is prone to overfitting and produces noisy pseudo-labels under extremely limited annotations [12,13,14]; (2) current models struggle to simultaneously capture high-level semantic distinctions among visually similar species and preserve fine-grained morphological details such as antennae, appendages, and boundaries, which are critical for accurate segmentation; and (3) the prevalent structural variability, pose diversity, and complex boundaries in zooplankton images further reduce pseudo-label reliability, thereby introducing noise and degrading model generalization performance.

To overcome these challenges, this paper proposes a semi-supervised semantic segmentation framework based on dual-teacher collaboration and multi-branch consistency, referred to as a consistency-driven dual-teacher model. An auxiliary teacher network with a heterogeneous architecture is introduced to collaborate with the primary teacher network, forming a complementary supervision mechanism that effectively alleviates overfitting under limited labeled data. On this basis, a three-branch prediction scheme comprising the primary teacher, auxiliary teacher, and a fusion branch is designed to jointly generate pseudo-labels. Furthermore, an adaptive consistency-based filtering mechanism is proposed to select high-quality pseudo-labels by comprehensively evaluating inter-branch prediction consistency and confidence scores, which are then used to supervise the student network. This mechanism effectively suppresses the influence of low-confidence pseudo-labels and enhances the stability and reliability of the pseudo-supervision process.

In addition, a pixel-level annotated zooplankton microscopy dataset containing multiple species and complex morphologies is constructed, and extensive experiments are conducted on this dataset. Experimental results demonstrate that the proposed method consistently outperforms existing state-of-the-art approaches for zooplankton microscopy image segmentation. The main contributions of this work are summarized as follows:

- A dual-teacher collaborative training framework is proposed, in which heterogeneous teacher networks provide complementary supervision to effectively alleviate overfitting in low-annotation scenarios;
- An adaptive pseudo-label filtering mechanism based on multi-branch consistency and confidence-weighted fusion is designed, significantly improving pseudo-label quality and segmentation performance;
- Extensive experiments on a self-constructed dataset demonstrate that the proposed method achieves superior performance under various annotation ratios, exhibiting enhanced robustness and generalization capability.

## 2. Related Work

### 2.1. Deep Learning-Based Semantic Segmentation Methods

Deep convolutional neural networks (CNNs) have driven rapid progress in semantic segmentation. For example, HRNet [15] improves segmentation accuracy by maintaining and fusing multi-resolution feature representations, while PSPNet [16] introduces a pyramid pooling module to enhance the modeling of multi-scale features and contextual information. However, convolution operators inherently rely on local receptive fields, which limits their spatial modeling capability to neighborhood structures. As a result, CNN-based methods exhibit intrinsic limitations in capturing long-range semantic dependencies and complex inter-class relationships.

In recent years, Transformer-based segmentation frameworks have demonstrated growing advantages over CNN backbones on multiple benchmarks, owing to the self-attention mechanism that enables global dependency modeling in feature space. Methods such as SETR [17] first adopt a pure Transformer as the encoder, Swin Transformer [18] introduces hierarchical window-based attention to achieve multi-scale feature representation, and Mask2Former [19] unifies semantic segmentation using a query-based formulation and achieves state-of-the-art performance on public datasets. Compared with CNN-based approaches, these methods are more effective in modeling global morphology and complex structural relationships.

Despite their success in natural scene understanding, Transformer-based methods still face notable challenges when applied to zooplankton microscopic image segmentation, where fine-grained structural details dominate. Extremely thin boundary features such as antennae and cilia rely heavily on precise local texture modeling, whereas the global semantic emphasis of Transformers may lead to boundary blurring. In addition, the high cost of pixel-level annotation in microscopy images introduces noisy and unstable pseudo-labels in semi-supervised settings, which further limits the training stability of Transformer-based models under limited annotations. Moreover, the statistical distributions of self-attention features differ substantially from those of convolutional features, making them difficult to directly align during pseudo-supervision fusion and increasing uncertainty in the learning process.

Therefore, achieving effective representation of complex structural morphologies while maintaining training robustness remains a critical challenge in microscopic image segmentation. This challenge also provides important motivation for the design of subsequent semi-supervised learning mechanisms and dual-teacher collaborative strategies.

### 2.2. Semi-Supervised Semantic Segmentation Methods

Due to the high cost of pixel-level annotation, semi-supervised semantic segmentation has emerged as a promising research direction for zooplankton image analysis, as it enables collaborative training using a small set of labeled data together with a large amount of unlabeled images. Existing approaches can generally be categorized into two main groups: pseudo-label generation methods [20,21,22] and consistency regularization-based methods [23,24,25].

Pseudo-label-based techniques [26,27,28] generate predicted labels for unlabeled images using a teacher model and select high-confidence predictions to supervise the training of a student model. Representative methods such as PseudoSeg [29] and ST++ [30] adopt dynamic pseudo-label updating strategies and have demonstrated strong performance. Consistency regularization methods [31,32,33], represented by teacher–student frameworks [34], aim to enforce prediction consistency under different input perturbations or model perturbations. Subsequent works, including AEL [35], CPS [36], and U^2^PL [14], further improve training pipelines, consistency objectives, and pseudo-label updating mechanisms, achieving promising results in domains such as remote sensing and medical imaging.

Despite these advances, most existing semi-supervised segmentation methods still rely on a single teacher network. In complex scenarios such as zooplankton microscopy images, a single model is prone to overfitting to limited labeled data, which may result in noisy or misleading pseudo-labels and ultimately degrade training effectiveness. Therefore, more robust frameworks are required that integrate multi-teacher collaborative mechanisms with refined consistency constraints, in order to improve pseudo-label quality and enhance model generalization capability.

To provide a clearer overview of existing semi-supervised semantic segmentation approaches and their limitations, we summarize representative methods in Table 1, highlighting their core strategies, strengths, and remaining challenges.

As observed, most existing approaches rely on a single-teacher paradigm or homogeneous backbone structures, which may limit supervisory diversity and robustness under extremely limited annotations. Moreover, many pseudo-label filtering mechanisms are based on static confidence thresholds without explicitly modeling multi-source structural consistency. These limitations motivate the proposed dual-teacher collaboration and consistency-guided pseudo-label filtering framework.

### 2.3. Multi-Teacher Models and Feature Fusion Strategies

To overcome the limitations of single-teacher architectures, recent studies in semi-supervised learning have explored multi-teacher frameworks to improve the diversity and reliability of pseudo-labels. Existing works mainly adopt multiple homogeneous teacher models and enhance pseudo-label quality by aggregating their predictions. For example, DMT [39] employs two independent teacher models to generate pseudo-labels and mutually guide the student network, effectively improving training stability. GTA [37] introduces an auxiliary network to refine teacher outputs and interacts with the student encoder through an exponential moving average (EMA) strategy, thereby reducing the risk of error propagation. In addition, most existing approaches rely on simple confidence-based filtering and consistency constraints [40,41], retaining only predictions that are both high-confidence and mutually consistent for training. However, such static and coarse-grained filtering strategies remain insufficient for handling the complex structures commonly observed in zooplankton microscopy images.

Motivated by these observations, this work builds upon the mean-teacher (MT) paradigm and proposes a consistency-driven semi-supervised semantic segmentation framework with a dual-teacher and multi-branch architecture. Specifically, two structurally complementary teacher models with differentiated feature extraction capabilities are introduced, and a multi-branch prediction scheme is constructed. An adaptive consistency mechanism is employed to fuse and filter semantic features at different levels, enabling the selection of high-quality pseudo-labels. This design effectively enhances segmentation accuracy and robustness in complex zooplankton microscopic image scenarios.

### 2.4. Intelligent Zooplankton Recognition Methods

**Figure 1:**
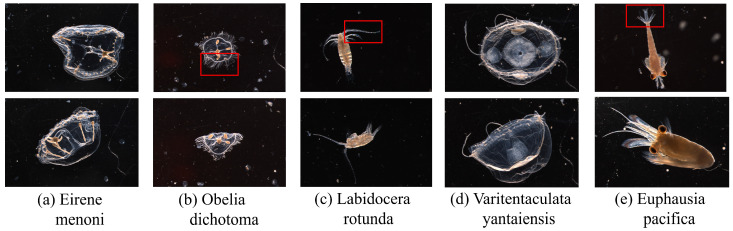
Intraspecific differences in zooplankton in different postures. Boxed areas highlight subtle morphological features prone to segmentation errors.

Zooplankton exhibit highly diverse morphologies, irregular boundaries, and intricate fine structures such as antennae and cilia, as illustrated in Figure 1. In the figure, each column (a–e) presents different poses of the same species, while different columns correspond to different zooplankton species. Owing to pose variations, individuals of the same species show pronounced intra-class variability, and commonly contain fine structures such as antennae and appendages. The boxed regions highlight subtle morphological details that are easily overlooked during segmentation, making accurate recognition a highly challenging task.

Traditional zooplankton recognition methods mainly rely on low-level features such as geometric shape descriptors and grayscale information [42,43,44]. These handcrafted features struggle to effectively capture the complex visual variations in zooplankton, often resulting in feature redundancy and limited classification accuracy. In recent years, the rapid development of deep learning techniques has led to significant breakthroughs in zooplankton recognition. Bureš et al. [45] applied transfer learning to freshwater zooplankton classification and achieved encouraging results; however, their approach exhibits limited generalization capability when extended to marine zooplankton. Li et al. [46] proposed a hybrid model combining DenseNet and YOLOv3 to improve recognition efficiency, but it still shows limitations in fine-grained species discrimination.

## 3. Materials and Methods

### 3.1. Motivation

**Figure 2:**
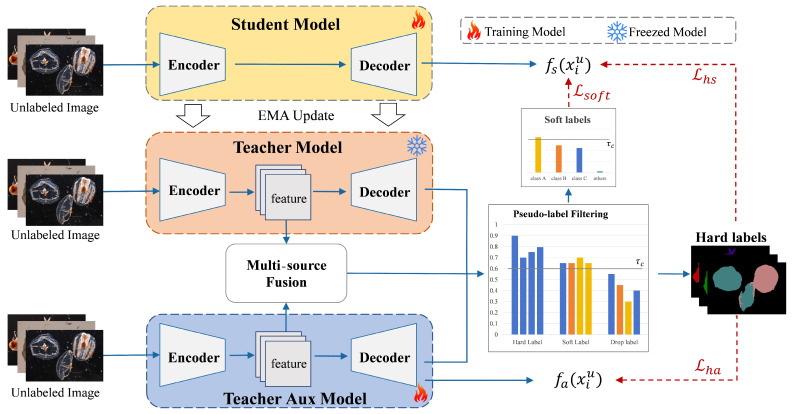
Overview of the dual-teacher semi-supervised segmentation framework. Different colors denote different semantic classes.

Semi-supervised segmentation methods exhibit notable limitations when applied to zooplankton microscopic images. Paradigms based on a single teacher network are prone to producing inaccurate pseudo-labels, which in turn weakens the effectiveness of pseudo-supervision. To address these issues, this paper proposes a semi-supervised semantic segmentation framework specifically designed for zooplankton microscopic images. The overall model architecture is illustrated in Figure 2, which presents only the unsupervised branch that processes unlabeled data. During training, two teacher models generate independent predictions for the same unlabeled image, and a third prediction is produced through feature fusion of the two teachers. Based on the consistency among the three predictions and their respective confidence scores, pseudo-labels are divided into hard-label regions and soft-label regions. Regions with high prediction consistency and high confidence are regarded as hard labels, while regions that are partially consistent yet contain rich semantic information are treated as soft labels. In the figure, the bar charts visualize the maximum confidence values of each prediction branch and their weighted fusion, where different colors denote different semantic classes. Soft labels are represented as full probability distributions derived from the fused predictions, which explicitly model inter-class uncertainty.

Built upon the classical teacher–student framework, the proposed method is enhanced by introducing two structurally complementary teacher models. Through feature fusion, the framework improves the representation of complex morphological structures. Furthermore, by incorporating a pseudo-label filtering mechanism driven by prediction consistency, the reliability of pseudo-supervision signals is substantially enhanced.

### 3.2. Heterogeneous Dual-Teacher Collaboration

In classical teacher–student frameworks, the teacher and student models typically adopt identical backbone network architectures. However, for the challenging task of zooplankton microscopic image segmentation, a single-architecture teacher model is prone to overfitting and struggles to simultaneously handle diverse target morphologies, ambiguous boundaries, and pose variations. To address this issue, this work introduces a heterogeneous dual-teacher design with distinct architectural differences, aiming to enhance the diversity and representational capacity of pseudo-supervision signals.

Specifically, ResNet-101 and ResNet-50 are employed as the backbone networks for the two teacher models, respectively. Owing to its deeper architecture and larger receptive field, ResNet-101 is more effective at capturing high-level semantic features and is well suited for modeling the global contours and overall morphology of zooplankton. In contrast, ResNet-50 is relatively lightweight and exhibits stronger capability in extracting local details, enabling more accurate capture of fine-grained structures such as boundaries, antennae, and appendages. By extracting features from the complementary perspectives of “global structure” and “local detail,” the two teachers provide diverse and complementary pseudo-supervision.

Such complementary modeling is particularly beneficial in complex microscopic scenes where individuals may be densely distributed, partially overlapped, or visually occluded. In these cases, global structural cues help preserve instance-level coherence even when certain regions are not clearly visible, while local-detail sensitivity improves discrimination along ambiguous or shared boundaries. Through collaborative supervision and subsequent consistency constraints, the framework reduces semantic confusion between adjacent individuals and mitigates the propagation of incorrect pseudo-labels in structurally challenging regions.

Moreover, both backbones benefit from well-established pretrained models and stable exponential moving average (EMA) update behavior, allowing them to deliver more reliable pseudo-labels during semi-supervised training. In comparison, although more heterogeneous CNN–Transformer combinations offer greater architectural diversity, their feature distributions are difficult to align during fusion. Under low-annotation conditions, this misalignment often leads to instability in the fusion branch and significant fluctuations in pseudo-label quality, resulting in inferior overall performance compared to the deep–shallow CNN pairing.

To preserve architectural heterogeneity and supervisory independence between the two teachers, gradient backpropagation is restricted exclusively to the student network. The parameters of the teacher models are updated using the exponential moving average of the student network weights, as formulated in Equation (1):

(1) $$\theta^{\prime }(t)=\rho \theta^{\prime }(t-1)+(1-\rho )\theta (t),$$

where $\theta^{\prime }(t)$ and θ(t) represent the parameters of the teacher and student models at iteration t, respectively, and ρ denotes the EMA hyperparameter satisfying ρ∈[0,1].

During the training process, the student model is optimized using two learning branches. The supervised branch employs ground-truth annotations from labeled images as supervision, whereas the unsupervised branch leverages pseudo-labels generated by the teacher models. For the supervised branch, the student model is trained using a standard pixel-wise cross-entropy loss function, as shown in Equation (2):

(2) $$L_{sup}=\frac{1}{H\times W}\sum_{i=1}^{H\times W}\ell_{ce}(f_{s}(x_{i}^{l}),y_{i}^{l}),$$

where $f_{s}(x_{i}^{l})$ represents the student network prediction for the i-th labeled image $x_{i}^{l}$, $y_{i}^{l}$ denotes the corresponding ground-truth annotation, and H × W is the total number of pixels in the predicted mask, where H and W denote the height and width of the predicted mask. For unlabeled images, the unsupervised loss $L_{unsup}$ is computed using the consistency-driven pseudo-label filtering mechanism between the dual teachers, which is detailed in Section 2.4. The overall training objective is defined in Equation (3):

(3) $$L=L_{sup}+L_{unsup}.$$

To enhance the reliability of pseudo-supervision signals, we do not adopt the strategy of extracting global and detailed features from different layers of a single backbone network. Although shallow and deep features differ in receptive fields, they still share the same parameter space, leading to strong feature correlations and making it difficult to form truly complementary perspectives. Moreover, shallow features are more sensitive to noise and background textures, and directly using them for pseudo-label generation may amplify prediction bias. In contrast, employing two structurally heterogeneous teacher models, namely ResNet-101 and ResNet-50, maintains independence in semantic abstraction and feature extraction bias. This design enables the two prediction branches to provide differentiated and robust supervision under complex morphological conditions, thereby further improving pseudo-label quality and the effectiveness of consistency constraints.

### 3.3. Attention-Guided Dual-Teacher Feature Fusion

To fully exploit the complementary advantages of the two teachers in terms of receptive field and semantic abstraction, we design a dual-branch fusion structure. The main branch extracts deep high-level semantic features $F_{main}\in R^{B\times C_{1}\times H\times W}$, while the auxiliary branch provides mid-level structural details $F_{aux}\in R^{B\times C_{2}\times H^{\prime }\times W^{\prime }}$, where B denotes the number of input images in a single forward propagation. These features are fused at higher stages to enhance the semantic representation of object categories while preserving fine-grained structural details in the target regions. To achieve effective cross-branch fusion, we first align the channel dimension and spatial scale of the auxiliary features. Specifically, the auxiliary features $F_{aux}$ are projected to match the channel dimension $C_{1}$ of the main features using a 1 × 1 convolution. Then, bilinear interpolation is applied to upsample them to the same spatial resolution. Finally, the two branches are concatenated along the channel dimension to form the fused feature representation:

(4) $$F_{fusion}=Concat(F_{main},Upsample({Conv}_{1\times 1}(F_{aux}))),$$

this operation facilitates a more coherent representation that combines high-level semantics with local structural details, supporting more robust segmentation of morphologically complex targets. To generate reliable pseudo-labels from the fused features while maintaining computational efficiency, we introduce a lightweight segmentation head following the fusion module. This design allows the network to produce supervisory signals with low overhead, ensuring that the fusion structure remains practical for semi-supervised training.

**Figure 3:**
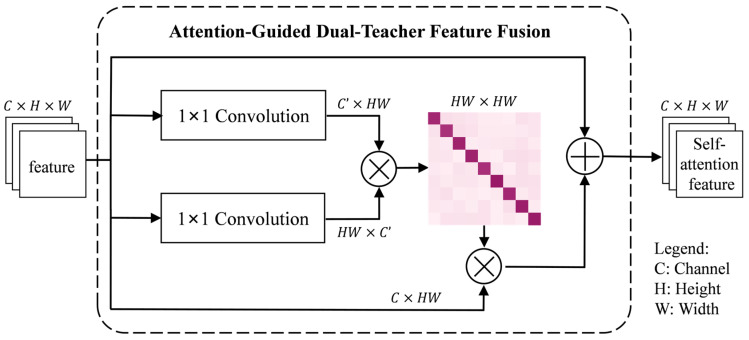
Architecture of Attention-Guided Dual-Teacher Feature Fusion module.

However, the inherent locality of convolution operations limits their ability to model long-range structural relationships in zooplankton microscopic images, which often contain highly diverse morphologies and complex boundary details. To address this limitation, we introduce a self-attention-based feature refinement module to enhance the consistency and contextual awareness of the fused features. The architecture of this module is illustrated in Figure 3, where H, W, C, and C′ denote the height, width, and channel number of the feature map, respectively. Considering the sensitivity of boundary information in zooplankton images as well as computational efficiency, the original fused feature is directly used as the value input in the attention mechanism, as formulated in Equation (5):

(5) $${\hat{F}}_{i}=\sum_{j=1}^{N}Softmax(\frac{\theta {\left(F_{i}\right)}^{T}\phi (F_{j})}{\sqrt{d_{k}}})F_{j},$$

specifically, $F_{i}$ denotes the fused feature at position i, and the output feature ${\hat{F}}_{i}$ is obtained by aggregating features weighted by attention scores. To extract spatial correlations between positions, we retain the linear projections θ and ϕ, enabling the attention mechanism to operate directly on the original feature structure and thus avoid information loss.

Finally, to enhance the consistency and structural coherence of the fused high-level semantic features, we introduce a residual connection to produce the final enhanced output:

(6) $$F=\hat{F}+F_{fusion}=\sum_{j=1}^{N}Softmax(\frac{\theta {\left(F_{i}\right)}^{T}\phi (F_{j})}{\sqrt{d_{k}}})F_{j}+F_{fusion}.$$

This design not only enhances the model’s ability to capture broader contextual relationships, but also preserves the structural integrity of the features and avoids redundant computation. Overall, the proposed fusion mechanism effectively improves segmentation performance under challenging conditions in zooplankton microscopic images—such as high intra-class variation, ambiguous boundaries, and complex morphologies—by integrating semantic and edge information with enhanced contextual representation.

### 3.4. Multi-Source Consistency-Guided Pseudo-Label Filtering

In semi-supervised segmentation tasks, the quality of pseudo-labels plays a critical role in model performance. A common strategy to denoise pseudo-labels is to down-weight or discard low-confidence regions. However, this approach typically relies on per-pixel confidence thresholds and often neglects the spatial consistency of pseudo-labels, resulting in structural fragmentation or semantic confusion. This issue is particularly pronounced in zooplankton segmentation scenarios, where a single zooplankton instance may be incorrectly divided into multiple classes, or adjacent but semantically different instances may be erroneously grouped together.

To enhance both structural consistency and semantic reliability of pseudo-labels, we propose a Multi-source Consistency-guided Pseudo-label Filtering (CPF) mechanism. This approach jointly considers the prediction divergence among multiple teacher models and the spatial structure of the outputs, aiming to improve the overall quality and completeness of pseudo-supervision signals. Specifically, to better control pseudo-label reliability, we divide the predictions into two categories: (1) Hard pseudo-labels with high confidence and consistent predictions across all branches, and (2) Soft pseudo-labels, where some prediction disagreements exist but the regions still contain potentially useful information. To clarify, hard pseudo-labels are represented as one-hot vectors corresponding to a single class, while soft pseudo-labels retain the full probability distribution output by the fused model. This allows the model to benefit from uncertainty-aware supervision, especially in ambiguous or boundary regions. By incorporating structural consistency into the filtering criteria, our method preserves reliable pseudo-labels while mining informative areas that might otherwise be discarded due to lower confidence.

We first perform weighted fusion of the predictions from three sources—main teacher branch, auxiliary teacher branch, and structure-aware branch—to integrate diverse discriminative cues. The fused probability distribution is defined as:

(7) $$P_{i,j}=\alpha \cdot P_{i,j}^{T}+\beta \cdot P_{i,j}^{A}+\gamma \cdot P_{i,j}^{F},$$

where $P_{i,j}\in R^{C}$ denotes the predicted class distribution at pixel position (i,j), and α, β, γ are fusion weights that satisfy α+β+γ=1.

Subsequently, to obtain reliable pseudo-labels with high confidence, a hard-label selection mechanism is established based on multi-source prediction consistency. When the three branches produce identical category predictions at the same spatial location and the fused confidence exceeds a dynamic threshold $\tau_{c}$ for the corresponding category, the pixel is classified as a hard pseudo-label region, as defined in Equations (8)–(10).

(8) $$I_{i,j}^{hard}=I(y_{i,j}^{T}{=y}_{i,j}^{A}=y_{i,j}^{F}),$$

(9) $$y_{i,j}^{h}=\left\{\begin{array}{c} y_{i,j}^{T},\;\;\;\mathrm{if}I_{i,j}^{hard}=1\;\mathrm{and}\;\max(P_{i,j})>\tau_{c}\\ 255,\;\;\;\mathrm{otherwise}\;\;\;\;\;\;\;\;\;\;\;\;\;\;\;\;\;\;\;\;\;\;\;\;\;\;\;\;\;\;\;\;\;\;\;\;\; \end{array}\right.,$$

(10) $$\tau_{c}\leftarrow \eta \tau_{c}+(1-\eta )\cdot max(Q_{0.7}(S_{c}),\tau_{min}),$$

where $S_{c}$ denotes the collection of historical confidence scores for class c, and $Q_{0.7}(S_{c})$ represents the 70th percentile of this distribution, the quantile value (0.7) is empirically selected to balance pseudo-label reliability and spatial coverage, as validated by the sensitivity analysis in Section 4.3.3. The threshold $\tau_{c}$ is dynamically updated based on the historical statistics using a momentum-based smoothing factor η.

Hard-label regions are regarded as high-quality pseudo-supervision signals and are used to train the student network with a standard cross-entropy loss, as formulated in Equation (11). In addition, since the auxiliary teacher model is structurally independent and cannot be updated via exponential moving average, hard pseudo-labels are further employed to explicitly supervise this auxiliary teacher in order to enhance its reliability, as expressed in Equation (12):

(11) $$L_{hs}=\frac{1}{\left|y_{i,j}^{h}\right|}\sum_{(i,j)\in y_{i,j}^{h}}\ell_{ce}(f_{s}(x_{i,j}^{u}),y_{i,j}^{h}),$$

(12) $$L_{ha}=\frac{1}{\left|y_{i,j}^{h}\right|}\sum_{(i,j)\in y_{i,j}^{h}}\ell_{ce}(f_{a}(x_{i,j}^{u}),y_{i,j}^{h}).$$

For regions that do not meet the consistency constraint, we retain a portion of pixels with relatively high confidence. To avoid sparse Soft regions during the early training stages or when confidence values are generally low, which could result in insufficient pseudo-supervision signals, we select the top k pixels with the highest confidence to fill in the density of the Soft regions, ensuring adequate optimization guidance. The Soft labels are used in the form of the fused probability distribution during training and provide flexible supervision with confidence-based weighting:

(13) $$y_{i,j,c}^{s}=\left\{(i,j)|I_{i,j}^{hard}=0,max(P_{i,j})>\tau_{c}\right\},$$

(14) $$y_{i,j,c}^{s}\leftarrow \left\{(i,j)|max(P_{i,j})\in T_{k}\right\},$$

where $T_{k}$ denotes the set of the top k pixels with the highest fused confidence values. The Soft labels directly supervise the student model’s prediction $f_{s}(x_{i,j}^{u})$ using the fused probabilities $P_{i,j}$, with the supervision weighted according to the confidence:

(15) $$L_{soft}=\frac{1}{\left|y_{i,j,c}^{s}\right|}\sum_{(i,j)\in y_{i,j,c}^{s}}\omega_{i,j}\cdot \ell_{kl}(f_{s}(x_{i,j}^{u}),y_{i,j,c}^{s}),$$

the weight term $\omega_{i,j}=\frac{max(P_{i,j})}{\mu }$ is used to emphasize the pseudo supervision signals with higher confidence. The final unsupervised loss for the model is computed as follows:

(16) $$L_{unsup}=L_{hs}+\lambda L_{soft},$$

where λ is the weight of Soft labels loss $L_{soft}$.

## 4. Results

### 4.1. Dataset

#### 4.1.1. Data Source and Basic Composition

In this study, a microscopic zooplankton image dataset, referred to as the Zooplankton-21 Microscopic Segmentation Dataset (ZMS-21), was constructed using samples provided by the Beihai Environmental Monitoring Center of the Ministry of Natural Resources. Due to the high cost and strong domain expertise required for pixel-level annotation of zooplankton microscopic images, there is currently a lack of publicly available datasets with high-quality pixel-wise annotations suitable for systematic evaluation of semi-supervised segmentation methods. Therefore, this work builds a dataset based on real monitoring samples to validate the effectiveness and robustness of the proposed method in practical application scenarios.

The dataset consists of 2507 high-resolution images covering 21 zooplankton species with diverse morphologies and poses. All images were acquired using an M205A microscope, with an original resolution of 2736 × 1024 pixels. To accommodate variations in specimen size and imaging conditions during acquisition, different zoom magnification settings were employed across samples, resulting in diverse imaging scales within the dataset. For subsequent model training, all images were standardized to a consistent spatial resolution following acquisition.

During image acquisition, no manual filtering was applied with respect to specimen pose, scale, or orientation. As a result, samples from the same category exhibit pronounced intra-class variability, while different categories share certain similarities in local structures and texture patterns. In terms of class distribution, the dataset shows a moderately imbalanced characteristic: classes with relatively large sample sizes contain approximately 180–220 images, whereas those with fewer samples include about 60–90 images. This distribution is consistent with the natural abundance patterns of zooplankton populations observed in real-world marine ecological monitoring.

The ZMS-21 dataset is specifically designed to support semi-supervised segmentation tasks for marine zooplankton with complex morphologies under limited annotation conditions.

#### 4.1.2. Annotation Protocol and Consistency

**Figure 4:**
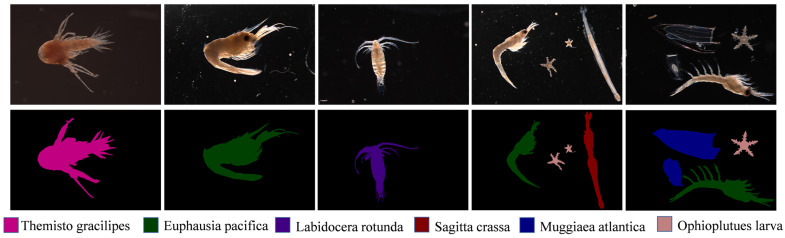
A Sample Image with Pixel-Level Annotations for Visualization.

All images were manually annotated at the pixel level under the guidance of domain experts. A unified annotation protocol was followed throughout the labeling process: all identifiable zooplankton individuals were annotated as foreground, while the surrounding water background, imaging noise, and non-biological particles were labeled as background. To preserve the true morphological characteristics of zooplankton, annotations covered not only the main body contours but also fine structures such as antennae, appendages, and cilia. An example of the pixel-level annotation is shown in Figure 4. For regions with ambiguous boundaries or low local contrast, annotators made judgments based on morphological continuity and structural characteristics, avoiding excessive simplification of object contours.

When multiple individuals were in contact or partially overlapping within an image, individual instances were distinguished primarily according to overall morphology and structural orientation to ensure spatial coherence of the foreground regions. For areas with poor imaging quality where reliable identification was not feasible, foreground labeling was intentionally avoided to reduce the influence of noisy annotations on model training. To assess annotation reliability, a subset of samples was randomly selected and independently annotated by different annotators following the same protocol, and consistency analysis was conducted. The results indicate high inter-annotator agreement in main body regions and major structural components, with discrepancies mainly occurring in extremely fine structures or boundary-ambiguous areas. Overall, the annotation quality is sufficient to support model training and performance evaluation.

#### 4.1.3. Data Split and Semi-Supervised Setup

In the experimental setup, the complete dataset was split into training and test sets at a ratio of 9:1. The test set was kept fixed across all experiments and used exclusively for performance evaluation. To simulate semi-supervised learning scenarios under varying degrees of annotation scarcity, the labeled samples in the training set were further divided into four proportions: 1/2, 1/4, 1/8, and 1/16. The remaining samples were treated as unlabeled data and jointly used during training.

This configuration enables a systematic evaluation of the stability and robustness of the proposed method under different supervision levels, and is particularly suitable for practical zooplankton microscopy image segmentation tasks, which are characterized by complex structures, rich fine-grained features, and high annotation costs.

### 4.2. Implementation Details

#### 4.2.1. Training Configuration and Experimental Setup

Following previous studies [47,48,49,50], ResNet-101 [51] pre-trained on ImageNet [52] was adopted as the backbone network and combined with a DeepLabv3+ [53] decoder. The model was optimized using stochastic gradient descent (SGD) with an initial learning rate of 0.001, a weight decay of 0.0005, and a momentum of 0.9. All input images were resized to 512 × 512 pixels using an aspect ratio–preserving strategy. Specifically, each image was first padded to a square resolution and subsequently scaled to the target size, ensuring consistent input representation across the dataset while maintaining the geometric integrity of fine morphological structures. The EMA update coefficient was set to 0.999. For multi-teacher branch prediction fusion, the fusion weights were set to 0.4, 0.3, and 0.3, respectively.

In soft pseudo-label selection, when the effective soft-label region accounted for less than 1% of the total unlabeled pixels, the top 10% of pixels with the highest prediction confidence were retained to ensure sufficient supervision. The loss weight for soft pseudo-labels was set to 0.5.

To further ensure experimental reproducibility, all experiments were conducted on a workstation equipped with 2 NVIDIA GeForce RTX 3090 GPUs (24 GB VRAM each). The operating system was Linux. The implementation was developed using Python 3.8 and PyTorch 1.4.0. Unless otherwise stated, models were trained for 60 epochs with a batch size of 16.

It is worth noting that although the ResNet family was proposed relatively early, its feature extraction capability, gradient stability, and training controllability have been extensively validated on large-scale datasets. As a result, ResNet remains one of the most widely used and stable backbone networks for semantic segmentation, and has been adopted by methods such as DeepLabv3+, CPS, and U^2^PL. Since the primary focus of this study lies in dual-teacher collaboration and pseudo-label filtering mechanisms, ResNet-101 and ResNet-50 were selected to construct heterogeneous teacher networks in order to ensure fair comparison with mainstream methods and to avoid performance bias introduced by backbone differences.

To ensure experimental reproducibility and assess statistical significance, each experiment was repeated five times using different fixed random seeds for dataset partitioning, weight initialization, and data augmentation. We report the mean and standard deviation for each method. Furthermore, a paired two-sided *t*-test was performed between the proposed method and the strongest competitor (DDFP) to confirm that the observed improvements are statistically meaningful.

#### 4.2.2. Runtime Efficiency and Deployment Feasibility

In practical marine ecological monitoring scenarios, computational efficiency and deployment feasibility are as important as segmentation accuracy. The proposed framework introduces a dual-teacher architecture and a multi-branch fusion mechanism during training, which increases computational overhead compared to single-teacher approaches.

It is important to clarify that the dual-teacher networks, feature fusion module, and consistency-based pseudo-label filtering mechanism are only utilized during the training phase. During inference, only the trained student network is retained for prediction, while both teacher networks and the pseudo-label filtering components are discarded. Therefore, the inference-time computational complexity of the proposed method is equivalent to that of a standard DeepLabv3+ model with a ResNet-101 backbone.

Under a 512 × 512 input resolution, the student model contains approximately 60–63 million parameters and requires approximately 160–180 GFLOPs per forward pass. On a modern high-end GPU platform, the average inference latency is approximately 22–30 ms per image (batch size = 1) after warm-up, corresponding to a throughput of approximately 33–45 frames per second (FPS).

Compared with the baseline GTA framework, the training-time computational overhead increases by approximately 2.0×–2.4× due to the additional teacher network and fusion branch. However, since these components are discarded during inference, the deployment cost remains identical to that of a single DeepLabv3+ model. This confirms that the proposed method remains practically deployable in real-time or near-real-time monitoring scenarios.

#### 4.2.3. Evaluation Metrics

Model performance was evaluated using a comprehensive set of segmentation metrics, including mean Intersection over Union (mIoU), Precision, Recall, F1-score, and Dice coefficient.

The mean Intersection over Union (mIoU) quantifies segmentation accuracy by measuring the overlap between predicted masks and ground-truth annotations. Precision is defined as the proportion of correctly predicted foreground pixels among all pixels predicted as foreground, reflecting the model’s ability to suppress false positives and avoid background misclassification. Recall represents the proportion of correctly detected foreground pixels among all ground-truth foreground pixels, indicating the model’s sensitivity to small or thin structures and its capacity to reduce missed detections. The F1-score is the harmonic mean of Precision and Recall, providing a balanced evaluation of detection performance under potential class imbalance conditions. The Dice coefficient quantifies the degree of overlap between predicted and ground-truth masks, and is particularly sensitive to small-scale structures and boundary regions.

Due to the presence of fine-grained appendages, antennae, and complex morphological variations in zooplankton images, Dice serves as an important complementary metric for assessing structural integrity preservation.

### 4.3. Ablations Studies

To further analyze the contribution of each component in our method, we conduct comprehensive ablation experiments on the plankton segmentation dataset under the 1/4-labeled setting using ResNet-101 as the backbone.

#### 4.3.1. Module Effectiveness Analysis

As shown in Table 2, we progressively evaluate the effectiveness of each component in the proposed framework. A model based solely on the Gentle Teacher Assistant (GTA) framework is adopted as the baseline for comparison. Key modules are incrementally integrated into the baseline model to assess their individual contributions. As reported in Table 2, the baseline model achieves an mIoU of 69.56%. Introducing the dual-teacher architecture (DT) results in a performance gain of +0.18%, validating the complementary benefits of multi-perspective guidance and redundant supervision. Further incorporating the attention-guided dual-teacher fusion (ADF) branch yields an additional improvement of +0.39%, indicating that the proposed multi-level feature interaction mechanism effectively enhances the representation of spatial details and global semantics. Finally, combining the consistency-guided pseudo-label filtering (CPF) mechanism boosts performance to 70.32%, demonstrating its critical role in improving pseudo-label quality by suppressing low-confidence and noisy predictions, which is particularly beneficial in complex biological background scenarios.

#### 4.3.2. Multi-Teacher Architecture Analysis

As shown in Table 3, we systematically investigate the impact of different teacher model configurations on segmentation performance. Experiments are conducted under the Gentle Teacher Assistant framework, with a single teacher network (ResNet-101) generating pseudo-labels as the baseline. On this basis, we first introduce a second teacher branch with the same architecture (ResNet-101). This configuration leads to a noticeable performance improvement, indicating that incorporating a multi-teacher mechanism can effectively enhance the stability and effectiveness of pseudo-supervision by alleviating overfitting to a single predictive perspective. A similar trend is observed when employing a homogeneous shallow teacher pair (ResNet-50 + ResNet-50), where the performance gain remains limited due to the lack of complementary feature representations across identical architectures. Further incorporating the ADF module into the homogeneous dual-teacher setting also leads to additional improvement, indicating that feature fusion is beneficial even when the teacher architectures are identical.

Furthermore, replacing the second teacher with a structurally heterogeneous network (ResNet-50) leads to a more pronounced performance improvement compared with the homogeneous dual-teacher setting. Notably, when compared under the same fusion condition, the heterogeneous teacher pair still outperforms its homogeneous counterpart, suggesting that the observed gain stems from complementary representation bias rather than simple architectural redundancy. This result confirms that heterogeneous teacher models provide complementary inductive biases in terms of receptive field range, levels of semantic abstraction, and feature representation patterns. Such architectural diversity offers multi-perspective criteria for pseudo-label generation, thereby yielding richer and more informative supervision during training.

Finally, the introduction of the attention-guided dual-teacher feature fusion module further improves segmentation performance. This outcome highlights the importance of cross-level and cross-branch feature integration, as aggregating multi-scale features from different teacher networks enables the model to more effectively capture fine structural details and contextual relationships—an advantage that is particularly critical for fine-grained animal image segmentation tasks.

#### 4.3.3. Sensitivity to Quantile Threshold

This study further investigates the impact of the quantile value used for the dynamic threshold in hard pseudo-label selection. As shown in Table 4, setting the quantile to 0.7 achieves the best balance between label reliability and spatial coverage.

When the quantile is set too low (e.g., 0.5), more pixels are retained for training; however, the relaxed threshold allows uncertain predictions and noisy labels to be incorporated into the student model, ultimately degrading overall pseudo-label quality. In contrast, when the quantile is set too high (e.g., 0.9), the generated labels exhibit higher confidence but the number of effective pixels is drastically reduced. This not only weakens the overall supervision strength but also significantly slows down model convergence.

These results demonstrate that proper calibration of confidence thresholds is critical in semi-supervised learning frameworks. Adopting a moderate quantile threshold (Q = 0.7) effectively filters out low-quality labels while preserving a sufficient number of high-confidence regions, thereby ensuring stable and effective model optimization.

#### 4.3.4. Impact of Soft Region Density

As shown in Table 5, we evaluate the impact of different density levels in the Top-k soft label selection mechanism. The results show that selecting the top 10% most confident pixels per image yields the best performance under the 1/4 supervision ratio.

When the density threshold is set too low (e.g., 5% or 1%), the selected regions exhibit very high accuracy but are overly sparse, resulting in insufficient supervision strength to effectively guide model training. In contrast, increasing the threshold to 20% introduces a larger number of pseudo-labels, but also incorporates many low-confidence noisy labels, which may cause semantic drift and unstable gradient updates, ultimately leading to performance degradation.

These results confirm that the Top-k strategy plays a critical role in balancing soft-label density and label quality. Adopting k = 10% ensures that pseudo-labels are sufficiently dense to provide effective learning signals while keeping noise at a controllable level. Therefore, this configuration is used as the default setting in this study.

### 4.4. Comparison with SOTAS

To comprehensively validate the effectiveness of the proposed method, we conduct comparative experiments against multiple state-of-the-art semi-supervised semantic segmentation approaches under a unified experimental setting. Although no public benchmark dataset currently exists for semi-supervised zooplankton microscopic image segmentation, the self-constructed dataset used in this study is built from real environmental sampling and imaging, and poses substantial challenges. Specifically, it exhibits pronounced inter-class similarity, blurred object boundaries, and high species diversity, providing a realistic and demanding evaluation platform for segmentation performance.

To ensure fair comparison, all methods are implemented using the DeepLabV3+ framework with a ResNet-101 backbone and evaluated under four labeling ratios: 1/2, 1/4, 1/8, and 1/16. The mean Intersection over Union (mIoU) is adopted as the primary evaluation metric, and model robustness is further analyzed by comparing performance across different supervision levels.

As reported in Table 6, under the four labeling ratios of 1/2, 1/4, 1/8, and 1/16, the proposed method achieves significant improvements of 16.04%, 21.64%, 22.64%, and 19.07%, respectively, over the fully supervised baseline (SupOnly). Notably, under the 1/8 labeled setting, our method with a ResNet-101 backbone outperforms the current best-performing method, DDFP, by 0.46% in terms of mIoU, demonstrating the superiority of the proposed framework and strategies for the challenging task of semi-supervised zooplankton microscopic image segmentation.

To further provide a more comprehensive evaluation of segmentation performance under the 1/4 labeled setting, additional quantitative metrics including Precision, Recall, F1-score, and Dice coefficient are reported in Table 7. These metrics offer complementary insights into boundary preservation capability, false-positive suppression, and fine-structure detection sensitivity beyond the region-overlap evaluation measured by mIoU.

To provide a more comprehensive understanding of the performance characteristics, the proposed framework is further evaluated across multiple annotation ratios and complementary evaluation metrics. As shown in Table 6 and Table 7, consistent performance gains are observed under all four supervision levels (1/16, 1/8, 1/4, and 1/2), indicating stable learning behavior across varying annotation budgets. In addition to mIoU, improvements are simultaneously reflected in Precision, Recall, Dice coefficient, and F1-score, suggesting that the observed performance trends are not confined to region-overlap measures but also extend to boundary preservation and fine-structure detection capability.

**Figure 5:**
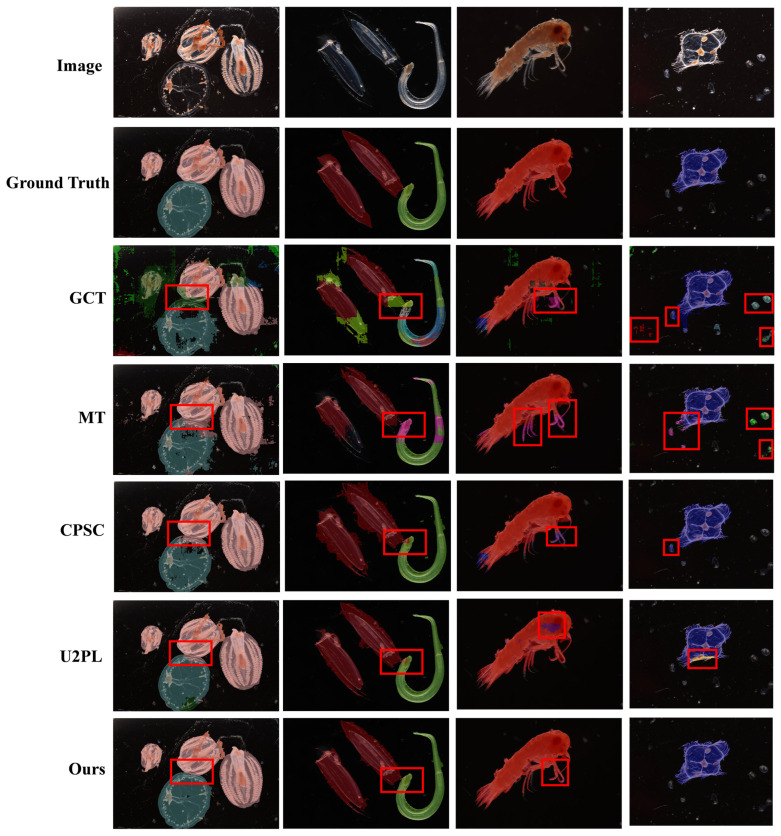
Visual comparison of different state-of-the-art methods on our plankton dataset. All models are trained under the 1/4 partition protocol. Red boxes highlight the ambiguous semantic boundaries between adjacent zooplankton individuals.

In addition, to provide a more intuitive comparison, Figure 5 visualizes the segmentation results of different methods on representative samples. The red boxes highlight ambiguous semantic boundaries between adjacent individuals, where existing methods tend to confuse overlapping structures. As highlighted in the zoomed-in regions of Figure 5, competing methods tend to incorrectly classify background impurities as foreground targets, whereas the proposed method suppresses such noisy responses and produces more compact segmentation masks. Overall, our method consistently produces more accurate object boundaries and better preserves structural integrity of individual instances, exhibiting improved spatial coherence and semantic reliability compared with other baseline approaches.

### 4.5. Statistical Significance Analysis

To evaluate the performance stability of the proposed method under different random initializations, statistical analysis is conducted on repeated experimental results under the 1/4 labeled setting.

All competing methods and the proposed approach were trained five times using identical data splits but different random seeds for network initialization and data shuffling. Table 8 reports the mean mIoU and standard deviation over five independent runs for the proposed method and the strongest baseline, DDFP.

The proposed method achieves a mean mIoU of 70.25% ± 0.12%, compared with 69.77% ± 0.10% obtained by DDFP.

Furthermore, a paired two-sided *t*-test was performed between the two methods across the five repeated runs. The resulting *p*-value is 0.004, which is below the commonly accepted significance level of 0.05. The mean performance improvement is 0.48%. This indicates that the observed performance improvement is statistically significant and unlikely to be caused by random initialization or stochastic optimization effects. These results indicate that the proposed method maintains stable segmentation performance across multiple independent runs with different random initializations.

## 5. Discussion

To more comprehensively evaluate the effectiveness of the proposed method for zooplankton microscopic image segmentation and to gain deeper insights into its advantages under different scenarios, we conduct a detailed analysis of the results reported in Table 6.

Under the 1/8 labeled data setting, the proposed method achieves an mIoU of 69.58%, representing a 22.64% improvement over the fully supervised baseline (SupOnly, 46.94%). Compared with the CPS method (50.46%), the performance gain reaches 19.12%. Even when compared with CPS+ (57.25%), our method improves mIoU by 12.33%. Relative to GTA (68.53%), the proposed approach achieves a 1.05% improvement. Compared with MT (56.19%), our method improves performance by 13.39%. In comparison with U^2^PL (64.08%), the proposed approach achieves a 5.50% gain. Finally, when compared with DDFP (69.12%), the proposed method improves mIoU by 0.46%.

As illustrated in Figure 5, the qualitative results are consistent with the quantitative improvements reported in Table 7.

In cases where individuals are densely arranged (e.g., columns (a) and (b)), existing methods often produce blurred boundaries or semantic confusion. In contrast, the proposed method accurately delineates each individual and preserves semantic independence.

Partially overlapped or occluded zooplankton individuals are frequently observed in microscopic images and represent a critical challenge for semantic segmentation. In such cases, blurred boundaries and shared appendages may cause semantic confusion between adjacent instances. As illustrated in the red box in Figure 5, existing single-teacher methods often exhibit boundary fragmentation or semantic merging when individuals are densely distributed. In contrast, the proposed dual-teacher framework maintains clearer structural separation.

This observation is consistent with the quantitative improvements reported in Table 7, where the proposed method achieves a Dice score of 82.59%, compared with 82.15% for DDFP and 82.06% for GTA.

The global-structure guidance from the deeper teacher improves instance-level coherence, while the local-detail sensitivity of the auxiliary teacher enhances boundary discrimination. The consistency-based filtering mechanism further mitigates noisy pseudo-label propagation in ambiguous regions.

Nevertheless, extreme occlusion or severe blur may still lead to consistent yet incorrect predictions from both teachers. In such rare cases, the framework may fail to fully separate heavily overlapped instances. Addressing extreme occlusion scenarios remains a potential direction for future research.

For samples with complex morphological structures and multiple appendages (e.g., column (c)), existing methods frequently fail to fully capture slender components, resulting in structural discontinuities.

This phenomenon is reflected in the lower Dice scores reported for GTA (82.06%) and DDFP (82.15%) compared with the proposed method (82.59%).

Moreover, in images with noisy backgrounds (e.g., column (d)), some methods tend to mis-segment non-biological particles as zooplankton.

The proposed method achieves a Precision of 84.48%, compared with 83.62% for DDFP, indicating improved suppression of false positives.

As shown in Table 7, the proposed method achieves an F1-score of 82.66%, outperforming DDFP (82.23%) by 0.43% and GTA (82.06%) by 0.59%.

These improvements across multiple complementary metrics further validate the effectiveness of the proposed dual-teacher collaboration and consistency-guided pseudo-label filtering strategy.

Overall, the quantitative comparisons in Table 6 and Table 7, together with the qualitative observations in Figure 5, indicate that the proposed method improves segmentation performance in scenarios involving densely distributed instances, diverse morphologies, and complex backgrounds.

## 6. Conclusions

This study addresses the challenge of limited pixel-level annotations in zooplankton microscopic image segmentation by proposing a novel semi-supervised segmentation framework. The proposed approach provides valuable insights for fine-grained animal image segmentation under scarce supervision and offers a scalable technical solution for algorithm development in intelligent marine ecological monitoring. By introducing a dual-teacher architecture with heterogeneous backbone networks, the framework enables complementary multi-perspective feature guidance. In combination with a structure-aware fusion branch and a consistency-guided pseudo-label filtering strategy, the quality of supervision signals under limited annotations is significantly enhanced. Extensive experiments conducted on a self-constructed zooplankton segmentation dataset demonstrate that the proposed method consistently outperforms existing mainstream approaches across different labeling ratios. These results validate the effectiveness of integrating multi-scale structural information and adaptive pseudo-label optimization in addressing the inherent challenges of complex morphologies and ambiguous boundaries in zooplankton images.

Nevertheless, several limitations remain. The segmentation performance may degrade for zooplankton samples with extreme morphological variations. In addition, the dual-teacher architecture and multi-branch fusion mechanism introduce additional computational overhead, which may limit deployment efficiency in resource-constrained scenarios. Furthermore, model performance is still influenced by the quality of initial annotations and class distribution, and mis-segmentation may occur in images with highly complex backgrounds or optical artifacts. Future work will explore lightweight dual-teacher designs to reduce computational cost, the incorporation of synthetic data augmentation and morphological priors to improve generalization to abnormal shapes, and the integration of multi-modal or temporal information to further enhance segmentation accuracy and practical applicability.

## Figures and Tables

**Table 1:** Comparative Analysis of Semi-Supervised Semantic Segmentation Methods.

Method	Year	Dataset Used	Core Technique	Performance Indicator	Advantages	Limitations
MT [34]	2017	CIFAR-10/SVHN/ImageNet	Teacher–student consistency using EMA weight averaging	4.35% error on SVHN with 250 labels	Improves prediction targets via weight averaging	Generated targets may be incorrect during training
CPS [36]	2021	Cityscapes/PASCAL VOC 2012	Cross Pseudo Supervision using two perturbed networks	Achieves state-of-the-art semi-supervised segmentation performance	Encourages prediction consistency across networks	Pseudo segmentation maps are unstable in early optimization stages
AEL [35]	2021	Cityscapes/PASCAL VOC 2012	Confidence bank + Adaptive Copy-Paste + Adaptive CutMix + Sampling + Re-weighting	74.28%, 75.83%, 77.90% (Cityscapes 1/32, 1/16, 1/8)	Improves performance on tail categories	Pseudo-label noise may still affect training performance
ST++ [30]	2022	Cityscapes/PASCAL VOC 2012	Self-training with Strong Data Augmentation and selective re-training	Outperforms previous methods across extensive settings	Decouples teacher–student predictions via SDA	Incorrect pseudo labels may accumulate during training
GTA-Seg [37]	2022	Cityscapes/PASCAL VOC 2012	Teaching Assistant network with EMA-based knowledge transfer	Shows competitive performance on benchmark datasets	Pseudo labels facilitate feature representation learning	Unreliable pseudo labels may lead to inaccurate mask prediction
U^2^PL [14]	2022	Cityscapes/PASCAL VOC 2012	Reliable/unreliable pixel separation + category-wise negative queue	Surpasses state-of-the-art alternatives	Utilizes ambiguous predictions as supervision	Directly using unreliable predictions as pseudo labels may degrade performance
DDFP [38]	2024	Cityscapes/PASCAL VOC 2012	Density-descending feature perturbation via normalizing flow estimator	Achieves state-of-the-art performance on both datasets	Encourages decision boundary exploration in low-density regions	Feature density estimation introduces additional training complexity

**Table 2:** Results are presented under the 1/4 partition to assess the effectiveness of individual components.

Method	DT	ADF	CPF	mIoU
baseline ^1^	×	×	×	69.56
baseline + DT	√	×	×	69.68
baseline + ADF	×	√	×	69.74
baseline + CPF	×	×	√	69.91
baseline + ADF + CPF	×	√	√	70.16
baseline + DT + ADF	√	√	×	70.13
baseline + DT + ADF + CPF	√	√	√	**70.32**

^1^ The baseline model is trained using the GTA framework. DT: Dual-teacher architecture. ADF: Attention-Guided Dual-Teacher Feature Fusion. CPF: Consistency-based label filter. Note: Bold font indicates our proposed method and the best performance.

**Table 3:** Ablation study on multi-teacher structure and fusion module.

TeacherA	TeacherB	ADF	mIoU
$T_{101}$ ^1^	×	×	69.56
$T_{101}$	$T_{101}$	×	69.95
$T_{50}$	$T_{50}$	×	69.67
$T_{101}$	$T_{101}$	√	70.06
$T_{101}$	$T_{50}$ ^2^	×	70.10
$T_{101}$	$T_{50}$	√	**70.32**

^1^ ResNet-101, ^2^ ResNet-50.

**Table 4:** Effect of quantile threshold Q on pseudo-label filtering.

Metric	Q = 0.5	Q = 0.6	Q = 0.7	Q = 0.8
mIoU	58.41	69.25	**70.32**	65.34

**Table 5:** Soft region density analysis under 1/4 and 1/8 partition setting.

Ratio	k = 1	k = 5	k = 10	k = 20
1/4	66.61	68.93	**70.32**	59.08
1/8	65.17	**69.58**	68.20	57.62

**Table 6:** Experimental results on our dataset show that our approach performs competitively compared to other methods. Experiments are conducted on four partition settings: 1/16, 1/8, 1/4, and 1/2. CPS+ denotes the CPS method enhanced with CutMix for improved performance.

Method	1/16	1/8	1/4	1/2
SupOnly	45.73	46.94	48.68	57.88
CPS [36]	48.85	50.46	51.27	58.15
CPS+ [23]	55.43	57.25	58.20	60.32
GCT [54]	55.35	57.40	57.79	60.27
MT [34]	54.21	56.19	60.46	62.09
U^2^PL [14]	60.61	64.08	65.18	69.41
MGD [41]	62.88	64.03	66.94	70.29
GTA [37]	63.01	68.53	69.56	71.44
DDFP [38]	64.52	69.12	69.70	73.46
Ours	**64.80**	**69.58**	**70.32**	**73.92**

**Table 7:** Various evaluation metrics under 1/4 labeled setting.

Method	Precision	Recall	F1-Score	Dice
SupOnly	69.12	62.28	65.53	65.48
CPS [36]	71.42	64.71	67.88	67.79
U^2^PL [14]	80.05	77.81	78.91	78.92
GTA [37]	83.40	80.78	82.07	82.06
DDFP [38]	83.62	80.92	82.23	82.15
Ours	**84.48**	**80.93**	**82.66**	**82.59**

**Table 8:** Quantitative Comparison with State-of-the-Art Method.

Method	Mean mIoU (%)	Std (%)
DDFP [38]	69.77	0.10
Ours	**70.25**	**0.12**

## Data Availability

The original contributions presented in this study are included in the article. Further inquiries can be directed to the corresponding author.
